# Symptom Exacerbation and Adherence to Antiretroviral Therapy During the Menstrual Cycle: A Pilot Study

**DOI:** 10.1155/IDOG/2006/14869

**Published:** 2006-02-02

**Authors:** Pinaki N. Patel, Richard M. Grimes

**Affiliations:** ^1^School of Public Health, University of Texas Health Science Center at Houston, RAS 319, PO Box 20186, Houston, TX 77225, USA; ^2^College of Osteopathic Medicine, Kansas City University of Medicine and Biosciences, Kansas City, MO 64106-1453, USA

## Abstract

Our objective is to investigate the relationship between drug and menstrual cycle symptoms in HIV+ women receiving antiretroviral therapy (HAART). Interviews were done for 12 weeks with 54 HIV+ women receiving HAART. Patients were asked if they experienced any of 28 symptoms associated with HAART and the menstrual cycle and about HAART adherence. Weeks were divided into menstrual weeks (MWs), premenstrual weeks (PWs), and other weeks. Women reported more bloating (*P* = .02) and cramps (*P* = .001) during MWs. Skin problems (*P* = .08) and breast tenderness (*P* = .03) were experienced during PWs. Feeling tired/loss of energy (*P* = .05), joint pain (*P* = .02), and chills (*P* = .03) were higher in non-MW/PWs. Women were slightly less adherent during the MWs (89%) than PWs (94%) and other weeks (93%). Feeling sad or depressed (*P* = .01) was associated with nonadherence. Experiencing certain symptoms associated with both the menstrual cycle and HAART drugs was related with nonadherence.

## INTRODUCTION

Use of multidrug antiretroviral therapy to suppress viral replication is the recommended approach to treatment of HIV infected patients who meet certain clinical criteria. This has been demonstrated to reduce HIV-related morbidity and mortality. The current treatment guidelines suggest that patients be treated with highly active antiretroviral therapy (HAART), utilizing 3-4 drugs of at least two different classes [1, 2].

However, this treatment requires strict adherence in order to successfully suppress the virus. Paterson showed that taking over 95% of the doses was necessary to maximally suppress viral replication [3]. Failure to suppress the viral load to very low levels has been associated with the development of drug resistant virus. However, antiretroviral regimens are difficult to follow on a daily basis due to meal restrictions, complex dosing schedules, and side effects. Many studies have been conducted to try to develop predictors of adherence and nonadherence to antiretroviral regimens.

Factors associated with adherence include availability of social support systems, patients being able to fit their medications into their daily routine, and the patient's understanding that poor adherence leads to resistance. Other factors include understanding the importance of taking all medication doses, feeling comfortable taking medications in front of other people, having significant others who are knowledgeable and agree with the treatment regimen as well as keeping scheduled clinic appointments [2–4] .

Factors that contribute to poor adherence include complex treatment regimens, large numbers of pills or high frequency of dosing, a poor doctor-patient relationship, substance use, depression, and patient beliefs as well as food restrictions [5]. Moreover, main reasons for missing doses include forgetting, being too busy, being depressed, and having severe drug-related side effects [2]. The list of side effects from patients taking HAART include rash, dryness or itching, fatigue/loss of energy, difficulty falling or staying asleep, pain and numbness in the hands and feet, diarrhea, nausea and vomiting, trouble remembering, and changes in the way a patient's body may look due to weight gain or fat deposits [6]. Other side effects include anxiety, confusion, and vision problems [7].

It has also been noted that women are less adherent to antiretroviral regimens when compared to men [8, 9]. Previous reports have linked women being unable to adhere to their regimen to the fact that women are more likely than men to mention needing to care for others as a factor contributing to their busy schedules [10]. Gender differences have also been attributed to hormonal effects on the immune system [7–11].

Given that there is evidence that adherence is poorer in women and that side effects have been shown to affect the rate of adherence, it is necessary to examine factors that are unique to women that might impact side effects. The most obvious differences are biological. Among these are menstruation and the menstrual cycle. It is known that there can be a variety of symptoms at various times during the menstrual cycle. These symptoms include pain, water retention, negative affect, impaired concentration, behavioral change, arousal, and irritability. Other symptoms include anger, being anxious, crying, depression, difficulty sleeping or sleeping too much, mood swings, being stressed out, overwhelmed, sensitivity, sudden sadness, appetite changes, backlashes, bloating, breast tenderness, cramping, head-aches, tired/lack of energy, and weight gain [12, 13]. It should be noted that many of these symptoms overlap with the side effects of antiretroviral therapy and could be associated with forgetting to take or electing not to take one's medications.

Therefore, it seemed reasonable to examine whether adherence fluctuates throughout the menstrual cycle, whether HAART-related symptoms are experienced more frequently during various times in the cycle, and whether the symptoms affect adherence. An extensive review of the literature did not reveal any studies that have tested whether a woman's phase of the menstrual cycle intensifies the symptoms associated with HAART.

## SUBJECTS AND METHODS

Patients were recruited from three different clinics in Houston, Texas, USA, which are conducted by the Harris County Hospital District. These clinics only see indigent patients or those who are eligible for Medicaid. Women who were HIV positive, currently experiencing menstrual cycles, currently taking HAART, able to speak English and had a telephone were eligible for inclusion in the study. If the patient met the inclusion criteria and was interested in participation, she was then given an informed consent document to read and sign.

A drug recall questionnaire and the signs and symptoms checklist questionnaire (see Table 1) were given to the patients after they gave consent to participate in the study. These questionnaires asked the women to specify their adherence to their HAART regimens and which symptoms they experienced during the week prior to the beginning of the study. An additional question was asked to determine if the woman had had a menstrual period in the previous week. Basic demographic information was collected at this time.

Thereafter, telephone interviews asking the symptom, drug recall and menstrual period questions were conducted every week for up to twelve weeks. If patients were unable to be contacted on the day of followup, attempts to contact were made on the subsequent two days. If the patient was unable to be reached by the third day the patient's response for that given week was recorded as a nonresponse for that week. If patients were not able to be contacted between their last contact and the end of the twelve-week period, they were considered lost to follow up and their data were truncated at that point.

The drug recall questionnaire was also administered weekly for each antiretroviral drug the patient was currently taking, in order to determine which medications the patient missed as well as to quantify the number of doses missed for each drug. Once patients identified that they had started a period during the previous week, that week was designated as a “menstrual week” (MW). The week prior to that was designated as the “premenstrual week” (PW). All other weeks since the last period were designated as “nonmenstrual or premenstrual weeks” (non-MW/PWs).

Data were maintained in a SPSS 11.0 database and were analyzed using X^2^ test and Fisher's exact test for variables tested.

## RESULTS

A total of 54 patients who met the inclusion criteria were enrolled in the study. Thirteen possible weeks of information were collected (baseline and 12 phone calls); so there was a potential of collecting 702 weeks of menstrual cycle experience and drug adherence information. Fifteen patients were lost to followup sometime during the study and partial data were collected for these patients since inclusion criteria were still met. Two patients stopped taking HAART during the study. Data from the weeks prior to their stopping were used in the analysis. This reduced the number of possible weeks for which data could be collected to 525 weeks. Patients were able to be contacted during 380 or 72.4% of those weeks. Data were collected for 87 menstrual weeks, 67 premenstrual weeks, and 226 other weeks.

The sample consisted of 41 African-Americans (75.9%), 10 Hispanics (18.5%), and 3 Anglo (5.5%) women. The mean age was 36.9 years. Table 1 shows the distribution of symptoms by cycle phase during the three-month period among 54 patients enrolled in the study. It presents each symptom's occurrence in relation to each of the three cycle phases. Significant values (*P* < .05) were identified for 6 out of the 28 symptoms and mildly significant values (> .05 < .10) were identified for 2 of the 28 symptoms. As would be expected, women were more likely to experience feeling bloated (*P* = .02) and cramps (*P* = .001) during the menstrual week. Taste perversions seemed to be less likely to occur during the menstrual week (*P* = .08). Breast tenderness was more likely to occur during both the menstrual and premenstrual weeks (*P* = .03). Women were marginally more likely to report skin problems during the premenstrual week (*P* = .07). It was interesting that three of the symptoms—tiredness (*P* = .05), joint pain (*P* = .02), and experiencing chills (*P* = .03) were more likely to occur during non-MW/PWs.

The next portion of the analysis examined the relationship between drug adherence and cycle phase. Patients were queried as to whether they had missed any doses during that week. If the patient missed any doses they were deemed to be nonadherent for that week. This rather stringent criterion was utilized because Paterson had shown that adherence at less than 95% was associated with treatment failure [3]. All of the study subjects were taking dosages that required 2-3 administrations per day. Therefore, missing a single dose in a week meant that the patient's adherence was less than 95%. A chi-square analysis was conducted for this relationship. It was noted that women were less adherent during the menstrual week (89%) than during the premenstrual week (94%) and non-MW/PWs (93%). However, this difference was not statistically significant (see Table 2).

The final analysis examined the relationship between the list of symptoms in Table 1 and adherence to the HAART regimen. Only one of the symptoms was significant at the 0.05 level. Feeling sad or depressed was associated with nonadherence (*P* = .01).

## DISCUSSION

The data collected from this study shows that some of the symptoms that are associated with HAART seem to be exacerbated during a woman's menstrual cycle. What was surprising was the degree to which these symptoms were reported by these women. Of the 28 symptoms in the questionnaire, eleven were reported to occur in the women over 35% of the weeks regardless of the cycle phase. Another eight symptoms occurred in between 25 and 35 percent of the weeks irrespective of the phase of the cycle. Only eight symptoms occurred during less than 25% of the weeks. So, the failure to find a relationship between most of the symptoms and adherence may be due to the fact that these women experience high rates of symptomology at all times and any given symptom may not alter their adherence.

An additional problem with this study was the small sample of women that were studied. Because of this it was not possible to examine whether the actual HAART regimen that was taken by the women could be the cause of the symptoms. The women were taking a wide variety of regimens; some were new to HAART; others had been through a variety of regimens; some switched regimens in the middle of the study; other had non-HIV drugs that they were taking; and so forth. All of these considerations could have caused higher or lower numbers of weeks with symptoms. However, with a sample size of 54 women, it was impossible to have a large enough “N” to answer this question of any given regimen.

In this study, no relationship between drug adherence and the phase of the menstrual cycle was found. This was possibly due to the high levels of adherence that were reported by the women, with their reporting complete adherence in over 90% of the weeks. This was much higher than their primary care physician believed to be the case. However, the main reason that has been given for lack of adherence has been “forgetting.” Therefore, it is possible that the weekly reminders associated with the phone calls and/or knowing that someone was checking on their adherence altered their pill taking behavior.

This pilot study suggested some tendency to have higher rates of adherence at various different phases of the menstrual cycle. However, the high rates of reported adherence made it difficult to achieve statistical significance—possibly due to the weekly reminders. Therefore, it is suggested that the study might be repeated with a larger sample size and a different measure of adherence. If pill bottle caps which record when the bottle is opened are used (as was done in the Paterson study), or prescription refill data are used to measure adherence, the patients would not be constantly reminded to take their medications. This would be more like the true situation where there is no one reminding or checking up on the patient. This kind of study might better determine the effect of the menstrual cycle phase and the symptoms on adherence.

## Figures and Tables

**Table 1:** Frequency of symptoms by cycle phase among 54 study patients. NS= not significant.

Symptom	Menstrual week	Premenstrual week	Other weeks	Significance
	N = 87(%)	N = 67(%)	N = 226(%)	

Skin problems	17 (19.5)	24 (38.5)	63 (27.9)	*P* = .07
Feeling tired or loss of energy	42 (48.3)	38 (56.7)	143 (63.3)	*P* = .05
Difficulty falling asleep	42 (48.3)	33 (49.3)	111 (49.1)	NS
Difficulty staying asleep	37 (42.5)	34 (50.7)	103 (45.6)	NS
Pain/numbness in hands and feet	33 (37.9)	30 (44.8)	97 (42.9)	NS
Feeling like throwing up	25 (28.7)	22 (32.8)	74 (32.7)	NS
Vomiting	7 (8.0)	6 (9.0)	33 (14.6)	NS
Trouble remembering	44 (50.6)	30 (44.8)	106 (46.9)	NS
Pain in joints	30 (34.5)	24 (35.8)	112 (49.6)	*P* = .02
Pain in stomach	25 (28.7)	17 (25.4)	61 (27.0)	NS
Felt sad or depressed	31 (35.6)	25 (37.3)	92 (40.7)	NS
Muscle aches	41 (47.1)	28 (41.8)	117 (51.8)	NS
Felt nervous or anxious	30 (34.5)	27 (40.3)	84 (37.2)	NS
Diarrhea or loose bowel movement	23 (26.4)	19 (28.4)	60 (26.5)	NS
Headaches	47 (54.0)	29 (43.3)	129 (57.1)	NS
Hair loss or changes in way hair looks	18 (20.7)	15 (22.4)	43 (19.0)	NS
Problems with weight loss	8 (9.2)	10 (14.9)	38 (16.8)	NS
Problems with weight gain	20 (23.0)	20 (29.9)	61 (27.0)	NS
Problems with having sex	15 (17.2)	17 (25.4)	43 (19.0)	NS
Feeling dizzy or lightheaded	32 (36.8)	25 (37.3)	91 (40.3)	NS
Feeling bloated	46 (52.9)	28 (41.8)	79 (35.0)	*P* = .02
Fever	17 (19.5)	11 (16.4)	49 (21.7)	NS
Noticing that food tastes funny	13 (14.9)	16 (23.9)	61 (27.0)	*P* = .08
Swelling or tenderness in breasts	30 (34.5)	24 (35.8)	51 (22.6)	*P* = .03
Loss of appetite	19 (21.8)	13 (19.4)	55 (24.3)	NS
Chills	16 (18.4)	17 (20.9)	72 (31.9)	*P* = .03
Unusual sweating	26 (29.9)	22 (32.8)	72 (31.9)	NS
Cramps	48 (55.2)	20 (29.9)	77 (34.1)	*P* = .001

**Table 2:** Drug adherence during phases of the menstrual cycle. Chi square = 2.42; *P* > .05.

Noncompliance values	Week of period N (%)	Premenstrual week N (%)	Other weeks N (%)

Adherent	77 (88.5)	63 (94.0)	211 (93.4)
Nonadherent	10 (10.3)	4 (6.0)	15 (5.3)
Totals	87	67	226
